# The *ANKK1/DRD2* locus is a genomic substrate for affective priming and recognition of angry faces

**DOI:** 10.1002/brb3.405

**Published:** 2015-10-14

**Authors:** Alejandra Koeneke, Guillermo Ponce, Janet Hoenicka, Evelio Huertas

**Affiliations:** ^1^ Faculty of PsychologyComplutense University of MadridMadridSpain; ^2^ Instituto de Investigación Sanitaria Hospital 12 de OctubreMadridSpain; ^3^ Red de Trastornos Adictivos (RTA)MadridSpain; ^4^ Program in Rare and Genetic Diseases & IBV/CSIC Associated UnitCentro de Investigación Príncipe FelipeValenciaSpain; ^5^ Centro de Investigación Biomédica en Red de Salud Mental (CIBERSAM)ISCIIIMadridSpain; ^6^ School of Medicine at Ciudad RealUniversity of Castilla‐La ManchaCiudad RealSpain

**Keywords:** Affective priming, *ANKK1*, *DRD2*, endophenotype, facial expression recognition, psychopathy

## Abstract

**Introduction:**

Ankyrin repeat and kinase domain containing I (*ANKK1*) and dopamine D2 receptor (*DRD2*) genes have been associated with psychopathic traits in clinical samples. On the other hand, individuals high in psychopathy show reduced affective priming and deficits in facial expression recognition. We have hypothesized that these emotion‐related cognitive phenomena are associated with *Taq* IA (rs18000497) SNP (single nucleotide polymorphism) of the *ANKK1* gene and with C957T (rs6277) SNP of the *DRD2* gene.

**Methods:**

We performed a genetic association analysis in 94 self‐reported Caucasian healthy volunteers. The participants completed 144 trials of an affective priming task, in which primes and targets were emotional words. They also had to recognize 64 facial expressions of happiness, sadness, anger, and fear in an expression recognition task. Regarding the genetic analyses, *Taq* IA and C957T SNPs were genotyped.

**Results:**

We found that the C957T SNP TT genotype was associated with a stronger priming effect and a better recognition of angry expressions. No associations were found for the *Taq* IA SNP. In addition, *in silico* analysis demonstrated that C957T SNP is a marker of a regulatory sequence at the 5′ UTR of *ANKK1* gene, thus suggesting the involvement of the whole *ANKK1/DRD2* locus in cognitive–emotional processing.

**Conclusions:**

These results suggest that affective priming and recognition of angry facial expressions are endophenotypes that lie on the pathway between the *ANKK1/DRD2* locus and some deviant phenotypes.

## Introduction

Dopamine transmission has been implicated in the processing of emotionally salient information, specifically through D2 receptors (DRD2) (Hranilovic et al. 2008; Pecina et al. 2013). The gene that codes for dopamine D2 receptors (*DRD2*) maps to chromosome 11q22‐q23 along with the ankyrin repeat and kinase domain containing I gene (*ANKK1*) (Neville et al. 2004). The *ANKK1* gene encodes a putative kinase of unknown function which might also be connected with dopaminergic system functioning (Hoenicka et al. 2010; Garrido et al. 2011). Hence, the *ANKK1/DRD2* locus has been extensively associated with D2 receptors function and thus with dopamine‐related traits. In particular, within this locus is located the *Taq*IA single nucleotide polymorphism (SNP) (rs18000497) (Ponce et al. 2009). This SNP consists a glutamic acid (Cytosine, A2 allele) or lysine (Thymine, A1 allele) at residue 713 (E713K) in the ANKK1 amino acid sequence. The A1 allele has been previously associated with increased activity of striatal L‐amino acid decarboxylase, the final enzyme for dopamine synthesis (Laakso et al. 2005). Besides, *Taq*IA is in linkage disequilibrium with *DRD2* SNPs that affect *DRD2* splicing (Zhang et al. 2007). The *ANKK1/DRD2* locus has another SNP associated with brain dopaminergic function named C957T (rs6277). This SNP consists a synonymous polymorphic change within exon 7 of the *DRD2* gene (Duan et al. 2003) and has likewise a marked impact on D2 receptors availability (Hirvonen et al. 2004).

Significant neurobiological effects of the *ANKK1/DRD2* locus on cognitive and emotional processes have been reported. In healthy volunteers, *Taq*IA SNP has been associated with learning from errors (Klein et al. 2007), greater sensitivity to negative feedback (Althaus et al. 2009), attentional bias for affective facial expressions (Gong et al. 2013), extraversion (Smillie et al. 2010), etc. With respect to the C957T SNP, it has been associated with reward‐related impulsivity (White et al. 2009), fear conditioning and aversive priming (Huertas et al. 2010), heart‐rate changes under stress (Huertas et al. 2012), learning from negative outcomes (Frank et al. 2007, 2009), etc. From a clinical point of view, both *Taq*IA and C957T SNPs have been associated with a variety of psychiatric illnesses with deficiencies in dopaminergic functioning and/or emotional processing (Hoenicka et al. 2006; Ponce et al. 2009; Whitmer and Gotlib 2012).

Along these lines, Ponce et al. (2008) found an epistatic effect of *Taq*IA and C957T SNPs on the expression of psychopathic traits. Psychopathy has been associated, in turn, with alterations in some affective processes where the dopaminergic system plays a key role, such as affective priming (Blair et al. 2006) and emotional expression recognition (see Dawel et al. 2012; Marsh and Blair 2008, for meta‐analytic reviews). Therefore, we hypothesized here that these two emotion‐related cognitive phenomena are linked to *Taq*IA and C957T SNPs.

Affective priming has been extensively used to examine the automatic affective processing (Fazio 2001; Klauer and Musch 2003) and refers to the fact that the time needed to evaluate a target item is shorter when it is preceded by an affectively congruent prime than when it is preceded by an affectively incongruent prime. Recognition of facial expressions plays a key role in the communication of emotions, and is subjected to both environmental and genetic influences (Ekman 1993; Lau et al. 2009; Zhu et al. 2012). Both phenomena have been related to various disorders where affective processing plays a relevant role (e.g., Demenescu et al. 2010; Hooker et al. 2011; LeMoult et al. 2012), suggesting that they could be endophenotypes underlying deviant phenotypes.

In a sample of healthy Spanish volunteers, we conducted a genetic association study of *ANKK1 Taq*IA and *DRD2* C957T SNPs with affective priming and facial expression recognition tasks.

## Materials and Methods

### Participants

Ninety‐four self‐reported Caucasian volunteers (56.4% men), aged 18–30 years (M* *=* *21.97, SD = 3.24), participated in this study. Eighty‐three were undergraduate students at the Complutense University of Madrid and 11 were graduates. The study received approval from the local ethics committees of the Complutense University of Madrid (Faculty of Psychology) and from the Hospital 12 de Octubre (Madrid). All participants consented to the experimental procedures and the collection of saliva for DNA analysis, and signed informed consent documents.

### Apparatus and stimuli

Stimuli were presented on a 15‐inch monitor, positioned approximately 70 cm in front of the subject. The software was developed by the Technical Service of the Complutense University of Madrid (Faculty of Psychology).

For the affective priming task, 144 prime–target pairs were presented to each participant. Primes were positive, negative, and neutral words, and targets were positive and negative words. All these words (nouns, adjectives, and verbs) were selected from the Spanish adaptation of ANEW (Affective Norms for English Words) (Redondo et al. 2007), which provide normative ratings for each word on valence and arousal, among other dimensions. The mean valence of the positive, negative, and neutral words was 7.8, 1.7, and 4.9, respectively, on a scale from 1 to 9. The differences between the three means were significant (positive–negative: *t *=* *161.09, df = 118.26, *P *< 0.001; positive–neutral: *t *=* *55.12, df = 111.86, *P *< 0.001; negative–neutral: *t *=* *73.32, df = 68.35, *P *<* *0.001). The mean arousal of the positive, negative, and neutral words was 6.0, 6.3, and 4.4, respectively. Words were written in capital letters and were presented in font Arial Black 25.

The task consisted of an initial training phase (24 trials) followed by a testing phase (120 trials). In the testing phase, 40 prime–target pairs of affectively congruent words (20 positive–positive and 20 negative–negative), 40 pairs of affectively incongruent words (20 negative–positive and 20 positive–negative), and 40 control pairs (20 neutral–positive and 20 neutral–negative) were presented. The concrete prime word that paired with each target word varied pseudorandomly. To evaluate the target word, the participant pressed one of the two keys on the computer keyboard, to which labels had been adhered with the signs “+” and “−” and which were counterbalanced among participants.

For the expression recognition task, 64 face photographs obtained in color were used, 16 for each expression (happiness, sadness, anger, and fear). The faces corresponded to women and men similar to participants in age and physical features. They had been typified at the Complutense University of Madrid (López‐Coira and Huertas 1997) and classified on the corresponding expression for 60–100% of participants in the norm group. The aim of this variation was that the expressions had a range of ambiguity similar to that of everyday life. On a scale from 1 to 10, the mean expression intensity was 6.4 for happiness, 6.2 for anger, 6.5 for fear, and 6.2 for sadness. On the screen, photographs were 400 pixels wide and 600 pixels high. Under each photograph, four buttons (132 pixels wide and 38 pixels high) appeared. They were placed horizontally in line, and each one had the name of one of the four emotions written inside. The participant had to click on the corresponding button with the pointer of the computer mouse.

### Procedure

First, the participant completed the affective priming task. He or she was asked to classify the second word of each pair as positive or negative by pressing on the corresponding key, while ignoring the first word, and to respond as quickly and accurately as possible. After completing the training phase, the participant went on to the testing phase, which consisted of two 60‐trial blocks separated by a 2‐min rest.

Each trial began with a fixation cross for 300 msec. The prime word was then presented for 150 msec and, immediately afterward, appeared the target word, which disappeared when the participant responded or after 3 sec. This prime–target SOA (stimuli onset asynchrony) of 150 msec was chosen based on findings that the activation curve of affective priming has a maximum around this SOA (Hermans et al. 2001). RT (response time) was recorded from the onset of the target. The intertrial interval pseudorandomly ranged between 2.7 and 3.3 sec. The order of presentation of the targets was also pseudorandomized for each subject and adjusted so that no more than three targets of the same valence occurred after one another.

Next the expression recognition task was done. The participant was explained that he or she would see a series of faces showing different emotions (happiness, sadness, anger, or fear) and that he or she had to click on the button corresponding to the emotion expressed by the face that appeared in this trial. The participant was warned that expressions would sometimes not be evident, but nonetheless he or she should attempt to respond in any case. He or she was also told to respond as quickly and accurately as possible, and to be guided by first impressions. The 64 photographs were then presented, each once. The trial started with a fixation cross for 500 msec. Immediately afterward, a face and the four buttons with the emotion labels appeared and remained until the participant clicked on one of the buttons or until 5000 msec had elapsed. The photographs appeared in pseudorandom order. The intertrial interval varied pseudorandomly between 2 and 3 sec. Once the participant had finished this phase, a saliva sample was taken.

### Genotyping

DNA from saliva was collected and extracted using the Oragene kit following the manufacturer's instructions (DNA Genotek, Ottawa, ON, Canada). Genotyping of *ANKK1 Taq*IA and *DRD2* C957T SNPs was performed as described previously (Ponce et al. 2008). The resulting genotypes for *Taq*IA were clustered according to the presence of at least one A1 allele (A1+ genotype: heterozygous; A1− genotype: homozygous for the A2 allele). C957T genotypes were grouped by assuming a recessive model for the T957 allele: homozygous individuals for the T allele versus heterozygous and homozygous individuals for the C allele. HWE (Hardy–Weinberg equilibrium) was also determined: http://www.oege.org/software/hwe-mr-calc.shtml. No deviation from HWE was observed for C957T SNP (*χ* ^2^ = 1.12, df = 1, *P > *0.2909). In contrast, *Taq*IA SNP was not under HWE (*χ* ^2^ = 8.78, df = 1, *P < *0.0032), suggesting sample stratification, which could explain lack of equilibrium. Population stratification can be a problem for case–control association studies. However, our work design included only one sample of healthy participants. A low degree of *Taq*IA‐to‐C957T LD (linkage disequilibrium) has been previously found in the Spanish population (*D*′: 0.58, *r* ^2^: 0.14) (Ponce et al. 2008).

### Statistical analysis

For affective priming, the dependent variable was the evaluation RT in test trials. Two participants were excluded from analysis because their RTs were 3 SDs above the grand mean. Trials with no response or with incorrect responses (8.75%) were also excluded. Median RTs in all six conditions were computed for each participant to correct for potential outliers. For emotional expression recognition, the dependent variable was the number of correct recognition responses to each facial expression. One participant was excluded from analysis because the total number of correct answers was 3 SD below the grand mean. Trials with no response (1.34%) were also excluded.

Affective priming data were analyzed using mixed design ANOVAs (analyses of variance), one per SNP, by means of the Statistical Package for the Social Sciences of International Business Machines (IBM SPSS Statistics 19 for Windows, SPSS Inc., Chicago, IL), with prime and target as within‐subjects factors and genotype as a between‐subjects factor. The genotype × prime × target interaction was examined by post hoc comparisons using repeated‐measures ANOVAs. Differences between genotypes in the evaluation of both positive and negative targets when the prime was neutral were examined by univariate ANOVAs. Differences between genotypes in expression recognition were examined by univariate ANOVAs, one per expression and SNP. Post hoc comparisons were analyzed using also univariate ANOVAs. Greenhouse–Geisser correction was used when the Mauchly's sphericity test gave significant results.

Since previous findings have indicated gender differences in affective priming (Gohier et al. 2013) and in facial expression recognition (McClure 2000), gender was added to the model as a between‐subjects factor in all the ANOVA analyses to control for the gender effect. Bonferroni correction was used for multiple testing: as there were 10 hypothesized associations (two SNPs × five dependent variables—priming and the four facial expressions), the significance level was set at 0.005 for these 10 main comparisons.

## Results

### Participant characteristics and *ANKK1/DRD2* locus analysis

There were no significant differences in both mean ages and gender when comparing genotype subgroups (Table 1). None of the participants was homozygous for the A1 allele of the *Taq*IA *ANKK1* SNP.

**Table 1:** Participant genotypes and characteristics

SNP	C957T			*Taq*IA	
Genotype	CC	CT	TT	A1−	A1+
*N* (%)	11 (11.7)	49 (52.1)	34 (36.2)	50 (53.2)	44 (46.8)
Gender: male/female	4/7	27/22	21/13	24/26	28/16
Age: mean (SD)	22.8 (3.4)	22.1 (3.1)	21.6 (3.4)	21.6 (3.1)	22.4 (3.4)

A1− genotype: homozygous for the A2 allele; A1+ genotype: heterozygous. Gender distribution did not significantly differ in either the C957T SNP genotypes (*χ* ^2^ = 2.171, df = 2, *P *=* *0.338) or *Taq*IA SNP genotypes (*χ* ^2^ = 2.315, df* *=* *1, *P *=* *0.149).

**Figure 1:**
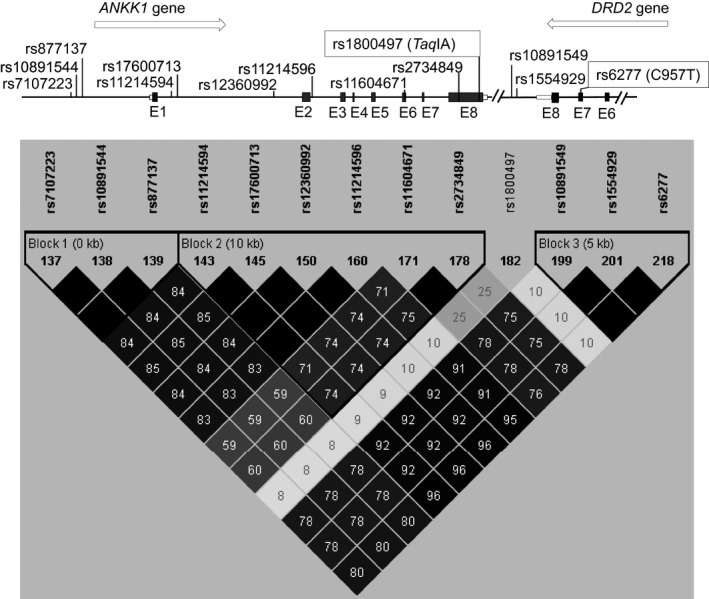
Pairwise tagging analysis plot of *DRD2* C957T SNP in a 500‐kb fragment of the locus in which *DRD2* and *ANKK1* are located. The analysis identified nine SNPs in two *ANKK1* haplotype blocks and two SNP at the *DRD2* 3′ region. Notably, the *Taq* IA SNP is not linked. Shading represents the magnitude and significance of pairwise LD, with a black‐to‐gray gradient reflecting higher to lower LD values. Black diamond without a number corresponds to *r* ^2^ values of 1.0. E: exon.

We have previously reported in the Caucasian HapMap sample that *Taq*IA *ANKK1* SNP is a marker of *DRD2* functional variants (Hoenicka et al. 2010). Here, since *ANKK1* and *DRD2* are overlapping genes at their 3′ untranslated regions (3′ UTR) (Hoenicka et al. 2010), we performed a pairwise tagging analysis in the same sample focusing in the C957T *DRD2* SNP (Haploview software version 4.2; Whitehead Institute for Biomedical Research; http://www.broad.mit.edu/mpg/haploview/index.php). We found that C957T *DRD2* SNP is in strong linkage disequilibrium with nine SNPs located in two *ANKK1* haplotype blocks (Fig. 1). Block 1 contained three SNPs located at *ANKK1* 5′ UTR in a transcription factors binding region (http://genome.ucsc.edu/ENCODE/), whereas Block 2 included four SNPs located at *ANKK1* introns 1 and 2, as well as rs11604671 and rs2734849, which cause an amino acid change in the ANKK1 protein. These data indicate that C957T SNP is also a marker of *ANKK1* expression variants. Therefore, *Taq*IA and C957T SNPs are markers of both *ANKK1* and *DRD2* genes functional variations. Consequently, we propose the *ANKK1/DRD2* locus to be a candidate functional unit, rather than its genes separately, for genetic association studies.

### Affective priming

To analyze the association of C957T SNP with affective priming, a genotype (CC, CT, TT) × prime (negative, positive) × target (negative, positive) ANOVA was performed. The prime × target interaction was significant (*F*_1,86_ = 9.801; *P* = 0.002). RT was longer when the prime and target were incongruent than when they were congruent, which indicates a global effect of affective priming. The genotype main effect was not significant (*F*_2,86_ = .284; *P *=* *0.754), but the genotype × prime × target interaction was significant (*F*_2,86_ = 6.589; *P *= 0.002; partial *η* ^2^ = 0.133), which reveals differences in the magnitude of affective priming between genotypes.

**Figure 2:**
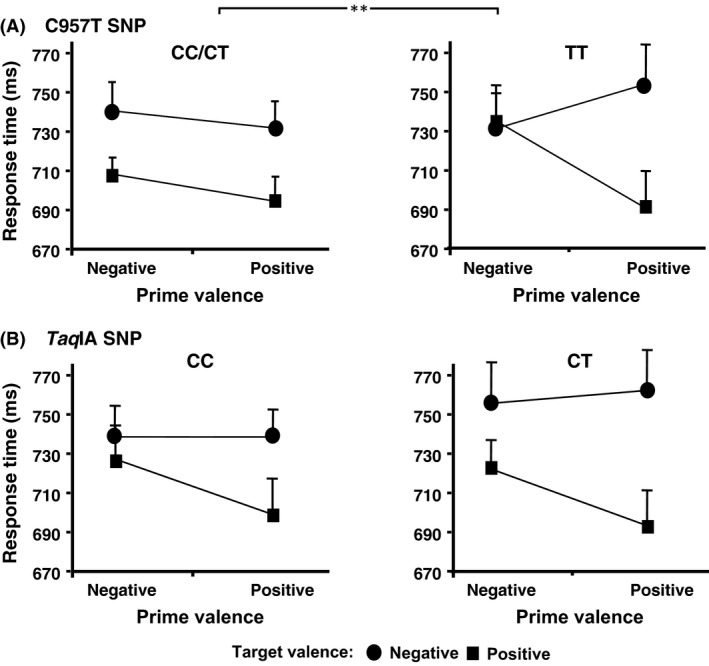
Response time for each prime–target condition in the affective priming task as a function of genotype. (A) Mean median response time in milliseconds, with standard error bars, relative to the target evaluation in each prime–target condition. The priming effect, as the prime × target interaction indicates, is stronger for TT genotype when compared with CC/CT genotype (**P =* *0.002). (B) Mean median response time relative to the target evaluation for each prime–target condition in the sample grouped according the *Taq* IA SNP genotypes when compared A1− versus A1+ carriers. No association was found for this SNP.

When CC and CT carriers were compared to each other, the genotype × prime × target interaction was not significant (*F*_1,55_ = 2.531; *P *=* *0.117), but it was significant when comparing genotypes CT and TT to each other (*F*_1,77_ = 12.691; *P *=* *0.001). When CT carriers were grouped with CC carriers, the genotype main effect was not significant (*F*_1,88_ = 0.094; *P* = 0.760), but the genotype × prime × target interaction was significant (*F*_1,88_ = 10.653; *P *= 0.002; partial *η* ^2^ = 0.108) (Fig. 2). The difference between the time needed to evaluate the target word under the prime–target affective incongruence conditions and the time required to make the same evaluation under the congruence conditions is higher for TT carriers when compared with CC/CT carriers. The prime × target interaction was not significant for carriers of genotypes CC/CT (*F*_1,57_ = 0.229; *P *=* *0.634), but was significant for carriers of genotype TT (*F*_1,31_ = 16.069; *P *<* *0.001).

Under the neutral priming conditions, significant RT differences between genotypes were not observed for positive targets (mean for genotype TT = 690.3; mean for genotypes CC/CT = 681.1; *F*_1,88_ = 0.004; *P *=* *0.952), or for negative targets (mean for genotype TT = 744.4; mean for genotypes CC/CT = 737.8; *F*_1,88_ = 0.105; *P *=* *0.746).

Regarding the *Taq*IA SNP, the genotype (CC, CT) × prime (negative, positive) × target (negative, positive) interaction was not significant (*F*_1,88_ = 0.131; *P *=* *0.718). Consequently no evidence of difference between genotypes in the magnitude of affective priming was obtained.

### Facial expression recognition

We first analyzed if there were differences among the three C957T SNP genotypes in the number of correctly classified faces for all four facial expressions. The ANOVAs gave significant differences between genotypes for the expression of anger (*F*_2,87_ = 6.882; *P *=* *0.002; partial *η* ^2^ = 0.137), but not for the expressions of fear (*F*_2,87_ = 0.083; *P *=* *0.920), happiness (*F*_2,87_ = 1.151; *P *= 0.321), or sadness (*F*_2,87_ = 1.557; *P *=* *0.217).

**Figure 3:**
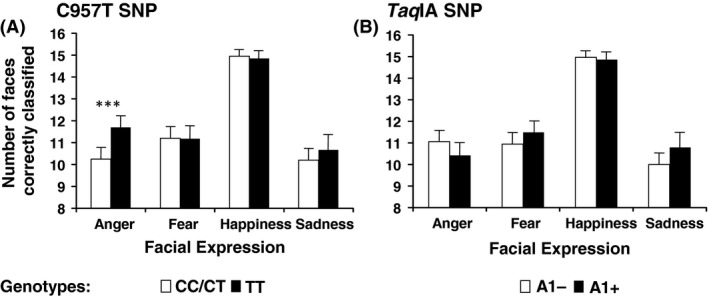
Correct recognitions of each facial expression as a function of genotype. (A) Mean number of facial expression images that were correctly identified by each genotype group of the C957T SNP, with standard error bars. (B) Mean number of facial expression images that were correctly identified by each genotype group of the *Taq* IA SNP. Sixteen faces of each expression were presented. *** indicates that the groups’ means are significantly different (*P *=* *0.001).

Carriers of TT genotype identified more faces showing the expression of anger than carriers of genotype CT (*F*_1,78_ = 8.306; *P *=* *0.005), while no significant difference was found between carriers of genotype CC and those of genotype CT (*F*_1,56_ = 1.969; *P *=* *0.166). When genotypes CC and CT were grouped (Fig. 3), the difference between the carriers of these two genotypes and carriers of genotype TT was significant (*F*_1,89_ = 11.980; *P *=* *0.001; partial *η* ^2^ = 0.119).

For the *Taq*IA SNP, no significant differences were obtained between genotypes in the number of correctly classified faces for the expressions of anger (*P *=* *0.197), fear (*P *=* *0.093), happiness (*P *=* *0.720), or sadness (*P *=* *0.110).

## Discussion

We found an association between the *ANKK1/DRD2* locus and both affective priming and recognition of angry expressions in healthy volunteers. Specifically, an association was found with C957T, but not with *Taq*IA SNP.

For affective priming, our C957T SNP data show that TT genotype carriers display more intense priming than the CC/CT carriers. There are two different, though not mutually exclusive, explanations of affective priming processing phenomena (Anderson 1984). Traditionally, it has been proposed that primes preactivate the representations of affectively congruent targets by spreading of activation through a network that link cognitive contents that share the same emotional valence (Fazio et al. 1986; Bargh et al. 1996; Fazio 2001). More recently, researchers have begun to conceptualize the affective priming in terms of conflict at the response stage of processing (Klinger et al. 2000). According to this interpretation, the prime would trigger a tendency to give a response in accordance with its valence; if both prime and target are incongruent, then this previous response tendency would interfere with the response relating to the target valence, but would facilitate it if they were congruent.

It does not seem reasonable to propose, in our case, that priming difference between genotypes is due to variations in processing the affective content of the words since we did not obtain significant differences in the time required to evaluate the target words when the prime was neutral. Rather, priming differences may be the result of variations at the response selection stage. Some previous data on C957T SNP would give support to this proposal. Genotype TT has been associated with reduced efficacy in inhibiting behavioral responses to a stop signal, and also with higher scores on self‐reported dysfunctional impulsivity (Colzato et al. 2010). In contrast, carriers of genotype CC have shown less trait impulsivity (Markett et al. 2014), greater efficacy in inhibiting unwanted action tendencies (Colzato et al. 2013), and higher cognitive controllability (Markett et al. 2010, 2011; Felten et al. 2013), which has the consequence of less everyday proneness to slips or errors (Markett et al. 2014). Thus, we suggest that, in our case, carriers of genotype TT might have more difficulty to prevent interference or facilitation of the response tendency generated by the affective content of the prime when they try to evaluate the affective content of the target. Likewise, Reuter et al. (2009a) found that the COMT Val158Met SNP, which is involved in dopamine degradation in the prefrontal cortex, was strongly associated with lexical decision latencies, but not with pure semantic processing, in a semantic priming task.

Interference in cognitive processing has also been studied in psychopathy, which as mentioned above, has been previously associated with genotype CC of C957T SNP (Ponce et al. 2008). In agreement with our results, research works involving fear‐potentiated startle have demonstrated that individuals high in psychopathy are less affected by peripheral information which competes with goal‐relevant information (Newman et al. 2010; Baskin‐Sommers and Newman 2013), although this peripheral information is affectively significant. In the same way, in our case, the allele C carriers could block more efficiently than the T homozygous the information relative to the affective valence of the prime during the target evaluation.

Regarding emotional expression recognition, carriers of genotype TT of C957T SNP show a better recognition of angry expressions than carriers of allele C. Nonetheless, no significant differences were found in the identification of expressions of fear, happiness, and sadness. This selective association with anger expression recognition is congruent with those theories which sustain that different neural systems exist that specialize, at least in part, in recognizing different types of emotional expressions (Kumfor et al. 2013). These systems would be related with the evolutionary history (Lawrence and Calder 2004). The processing of aggression signals, that is extremely relevant for survival, has been related with the dopaminergic system in various species (Lawrence et al. 2002) and is modulated by other relevant genetic and epigenetic biomarkers for social behavior like those related to oxytocin (Puglia et al. 2015). Furthermore, some data suggest that the same neural systems activated during the identification of a particular emotion in others also respond to the personal experience of this emotion (Assogna et al. 2008). In this direction, anger and aggression have also been related to the dopaminergic action (Swann 2003) and to genes related to the dopaminergic system (Rujescu et al. 2003; Reuter et al. 2009b).

Consistently with an involvement of the dopaminergic system in recognizing facial expressions of anger, Parkinson's disease patients have also shown selective impairments for the recognition of these expressions (Clark et al. 2008), particularly those who never received medication (Sprengelmeyer et al. 2003), or those who no longer received dopamine replacement therapy (Lawrence et al. 2007). Administering sulpiride, an antagonist of dopamine D2 receptors, has also been found to selectively deteriorate the ability to recognize expressions of anger in healthy individuals (Lawrence et al. 2002). In all these cases, recognition of some alternative emotions remains intact, which indicates that this is no general impairment in emotional expression recognition.

Therefore, in this study we found that T homozygous carriers of C957T SNP are more sensitive to the effect of emotional contents in some tasks, such as affective priming and recognition of facial expression of anger. Nevertheless, empirical evidence seems to indicate that CC carriers of C957T SNP show increased emotional processing in other circumstances. For instance, in healthy people, genotype CC has been associated with greater persistence of the skin conductance response to conditioned stimuli that predicts the appearance of an aversive stimulus (Huertas et al. 2012). It has also been associated with greater aversive–priming effect (Huertas et al. 2010), which is characterized by a positive bias in identifying threatening stimuli when an aversive stimulus occurs immediately before (Huertas 2012). Genotype CC has also been associated with neuroticism (Montag et al. 2012) and posttraumatic stress disorder (Voisey et al. 2009) that are conditions related to threat processing. These data altogether suggest that CC genotype carriers show less cognitive control of threatening information, which could lead to giving more autonomic responses to it. Therefore, the CC genotype itself might potentiate the activity of specific brain systems related to response to threats. However, this would not imply higher sensitivity to the affective content which confers emotional color to words, images, etc., if this content is not linked to threats to oneself.

Affective priming and emotional expression recognition share some encephalic structures, such as the ventral striatum and the prefrontal cortex (van den Bulk et al. 2013; Suslow et al. 2013) where dopaminergic D2 receptors are essential for dopaminergic signaling. C957T SNP has a marked impact on the well‐described variability in D2 dopamine receptors binding characteristics. The T allele of C957T SNP has been associated with lower mRNA stability and protein synthesis in vitro (Duan et al. 2003), and also with higher D2 dopamine receptors availability due to low density (Bmax activity) and high affinity (low K_d_ values) in the human striatum (Hirvonen et al. 2009a). In this scenario, the hypothesis of a net reduction of the D2 receptor function by reducing the dopamine tone in the striatum is feasible. In addition, the C957T SNP also regulates D2 dopamine receptors availability in both the human cortex and the thalamus in vivo (Hirvonen et al. 2009b). However, the observed pattern differed from that of the striatum, which may reflect distinctive roles of D2 receptors and dopamine in striatal and extrastriatal structures. In any case, several imaging studies have demonstrated the C957T effect upon D2 receptors in the brain (Hirvonen et al. 2004, 2009a,b). Moreover, C957T SNP is also a marker of a genomic sequence at the 5′ UTR of *ANKK1* gene. Although the mechanism by which this particular SNP might tag *ANKK1* transcriptional activity is presently unknown, this genetic link suggests a tight relationship between *ANKK1* and *DRD2* in the areas of the brain where D2 receptors are expressed. Functional studies of the *ANKK1* expression and its relation with the dopamine D2 receptors density could shed light into the investigation of the molecular mechanisms underlying pathways between the *ANKK1/DRD2* locus and psychiatric disorders linked to dopamine function.

The limitations of this study are related to the nature of the genetic association studies. To validate our results, a study of independent healthy control samples, as well as populations of individuals with disorders related to the *ANKK1/DRD2* locus, is needed. This would clarify if affective priming and recognition of emotional expressions are endophenotypes related to such disorders. The analysis of *Taq*IA and C957T SNPs interaction would also help to clarify the role of *ANKK1/DRD2* locus upon cognitive–emotional processing deviations, which seems to be inherent to disorders like psychopathy.

In conclusion, we found in healthy volunteers that the TT genotype of the C957T SNP is associated with increased affective priming and improved recognition of angry expressions. Given that this SNP is a marker of both *ANKK1* and *DRD2* functional variants, we propose that the *ANKK1/DRD2* locus could play a pivotal role in mediating emotional processing endophenotypes in patients showing a dopaminergic dysfunction. Our data suggest that affective priming and recognition of emotional expressions would partially lie on the pathway between the *ANKK1/DRD2* locus and some deviant phenotypes. Further studies of the functionality of C957T on the *ANKK1/DRD2* locus are warranted.

## Conflict of Interest

None declared.
